# Use of High Resolution GC/MS for Obtaining Accuracy in Lipid Measurements

**DOI:** 10.6028/jres.093.065

**Published:** 1988-06-01

**Authors:** John Savory, Michael Kinter, David A. Herold, Judy C. Hundley, Michael R. Wills

**Affiliations:** Departments of Pathology, Biochemistry and Internal Medicine, University of Virginia, Charlottesville, VA 22901

## Introduction

Inaccuracy in clinical chemistry measurements is a serious problem in both service and research laboratories. It is only by having procedures of proven accuracy available that the clinical chemist can gain assurance that a working method is providing meaningful data. Analytical uncertainties occur in all areas of clinical chemistry and two examples of such problems in the area of lipid analysis are discussed here.

## Prostanoid Measurements

One excellent example in which difficulties with inaccuracy can be seen is in the measurement of prostanoids. Although these analyses are not yet routine clinical chemistry measurements, these metabolites of arachidonic acid are of great importance in a vast number of research studies. Disturbances of prostanoid production are seen in a wide variety of pathological conditions including diabetes mellitus, hypersensitivity and inflammation, cancer, reproductive problems, hypertension and cardiovascular disease. To emphasize the analytical problems associated with measuring prostanoids, a survey of the literature reveals an incredible spread of reported reference (normal) ranges for various prostanoids in human plasma. For example, reported reference ranges for 6-keto-PGF_lα_ are 0.4–5.0 pg/mL [1], 19–25 pg/mL [2], 160–228 pg/mL [3] and 1500–1700 pg/mL [4].

We have applied gas chromatography/mass spectrometry (GC/MS) to the measurement of prostanoids in plasma and in urine. Our procedure initially used an initial extraction, clean-up by high performance thin layer chromatography [1], derivatization, and detection by GC/MS at low or unit mass resolution using negative ion chemical ionization. Using this technique, assays of marginal performance were developed for the measurement of PGE_2_, 6-keto-PGF_lα_, PGF_2α_, and TxB_2_ in plasma. However, when urine was analyzed, there were many interferences due to the complex matrix and reliable analytical data could not be produced.

The purpose of the present investigation was to reduce these potential interferences from biological specimens, particularly urine, by applying stable isotope dilution with quantitation by capillary column gas chromatography and high resolution mass spectrometry. The use of high resolution mass spectrometry is notable because it provides the selectivity of detection previously obtained by extensive chemical clean-up. This selectivity of detection can result in lower limits of detection despite a reduction in absolute sensitivity since one source of noise, chemical noise, is reduced more rapidly.

## Materials and Methods

Deuterium-labeled internal standards (d_4_-PGE_2_ and d_4_-6-keto-PGF_1α_) were added to 10 mL of acidified urine (pH=2.5). Initial extraction of the prostanoids was by a C_18_ SEP-PAK extraction, elution and subsequent ethyl acetate extraction from the eluate. Derivatization involved methoximation followed by a novel scheme in which the pentafluorobenzyl esters were formed prior to a silylation step in which the t-butyldimethylsilyl esters were formed in an apparent exchange reaction. Treatment with N-methyl-N-(t-butyldimethylsilyl)tri-fluoroacetamide (MTBSTFA) to produce the t-butyldimethylsilyl esters also, of course, formed the t-butyldimethylsilyl ethers.

Gas Chromatography/Mass Spectrometry. The mass spectrometer used was a double focusing, reverse geometry instrument (Model 8230, Finnigan MAT) with a SpectroSystem 300 data system. The instrument was operated in the electron ionization mode using 70 eV electrons. Data were acquired in a selected ion monitoring experiment, monitoring *m/z* 666.441 and 670.466 for PGE_2_, and 798.537 and 802.503 for 6-keto-PGF_1α_ at a rate of 1 Hz. A DB-1 bonded phase fused silica capillary column, 20 m×0.32 mm, was used. Samples were injected on column at a temperature of 200 °C followed by a 15 °C/min ramp to 300 °C.

## Results and Discussion

Recoveries through the entire extraction and derivatization procedure were 70.6% for PGE_2_ and 64.4% for 6-keto-PGF_1α_. Quantitation at mass resolution of 10,000 eliminated all interferences in the PGE_2_ chromatograms while a resolution of 1,000 was sufficient for the 6-keto-PGF_1α_ analysis with limits-of-detection of 50 pg/mL for each. For PGE_2_ a lower limit-of-detection was obtained at a mass resolution of 10,000 (50 pg/mL), than was obtained at a mass resolution of 1,000 (80 pg/mL), illustrating the selectivity of detection. The method was linear to 10 ng/mL. Spot urine specimens in normal volunteers ranged from 80–570 pg/mL PGE_2_ (n= 12) and 77–185 pg/mL 6-keto-PGF_1α_ (n=6). This method, although difficult and expensive, provides excellent analysis of PGE_2_ and 6-keto-PGF_1α_ in urine.

## Cholesterol

Recent studies on the use of serum cholesterol as a risk factor in atherosclerosis indicate a need for improved analytical performance in routine assays. The Laboratory Standardization Panel of the NIH National Cholesterol Education Program has set goals of <±3% for both bias and precision. Towards this end, the Panel has also established a definitive method, which uses isotope dilution mass spectrometry, and a reference method, which uses a colorimetric assay. There is, however, a continued need for the widespread availability of reference and pseudo-definitive methods with physical methods being preferred.

We have developed a GC/MS procedure for the measurement of serum cholesterol using a structural isomer, 7,(5α)-cholesten-3*β*-ol, as the internal standard which we used because the cost of a stable isotope labeled internal standard based assay was prohibitive due to the high concentration of endogenous cholesterol. In addition, the GC/MS measurement was made at a moderately high mass resolution (R=5000) to enhance the selectivity and ensure an interference- free assay. As was the case with the prostanoid measurements, high resolution mass spectrometry provided a highly selective measurement step. This selectivity, in this example, was used to eliminate interferences that were fundamental limitations to accuracy.

## Materials and Methods

Reagent and Standards. The cholesterol Standard Reference Material (SRM 911a) and Certified Human Serum (SRM 909) were obtained from the National Bureau of Standards (Gaithersburg, MD 20899). The latter had a certified cholesterol concentration of 143.1 mg/dL.

A 0.5 mL aliquot of serum and a 0.5 mL aliquot of the internal standard solution (7,(5α)-cholesten-3*β*-ol) were pipetted into a centrifuge tube using an automatic pipettor and the sample hydrolyzed in alcoholic KOH at 37 °C for 3 hours. The sample was then extracted and derivatized with BSTFA (bistrimethylsilytrifluoroacetamide) for 0.5 h at 60 °C. The samples were then evaporated to dryness and reconstituted in 500 *μ*L of tetradecane for analysis. A 211.4 mg/dL standard was also prepared by pipetting 0.5 mL of a standard cholesterol solution and 0.5 mL of the internal standard solution in a centrifuge tube, evaporating to dryness, and reconstituting the standard in 5 mL of methanol from which a 100 *μ*L portion was transferred for derivatization.

Gas chromatography/mass spectrometry analysis was performed in a similar manner to the prostanoid assay. Selected ion monitoring of *m/z* 458.394 at a rate of 4 Hz was carried out with GC conditions the same except that a 20 °C/min ramp was used.

## Results and Discussion

The 100 eV EI mass spectra of cholesterol and 7,(5α)-cholesten-3*β*-ol both showed molecular ions of large relative abundances, 70% and 100%, respectively. The molecular ion was therefore chosen as the ion to be monitored, maximizing our molecular specificity. These compounds were also quite easily separated using capillary column gas chromatography. Under our chromatographic conditions, the 7,(5α)-cholesten-3*β*-ol derivative eluted at 7:56 minutes and the cholesterol derivative eluted at 8:21 minutes. In addition, the signals obtained were quite large, allowing good counting statistics. No other chromatographic peaks were observed in the time window for which data were acquired, indicating good selectivity. A linear response, area cholesterol divided by area 7,(5α)-cholesten-3*β*-ol, was observed over a range of 0 to 1000 mg/dL. This linearity allowed the calculation of sample concentration using a response factor calculated from a single standard.

Concentration values for the Certified Human Serum determined by this method in a series of 7 analyses sets are shown in table 1. From these data the accuracy was excellent with an average concentration of 142.3 mg/dL indicating a bias of − 0.6%. The between-run precision, SD = 2.2 mg/dL, CV= 1.6%, is approaching the target precision of 2.5% for a serum cholesterol reference method. The in-run precision, estimated from the average of the ranges for the duplicate determinations, is also acceptable, SD = 2.3 mg/dL, CV=1.6%.

The performance characteristics of this assay are within the goals established for a serum cholesterol reference method. Good accuracy was to be expected because of the high molecular specificity of gas chromatography/high resolution mass spectrometry. This specificity ensures an interference-free assay eliminating interferences as a fundamental limitation to accuracy.

A GC/MS reference method for the measurement of serum cholesterol using a structural isomer as an internal standard has been developed. The advantages of this method are the cost-effectiveness of using an unlabeled internal standard and the specificity of using gas chromatography/high resolution mass spectrometry. This assay will allow the establishment of reference serum cholesterol assays in laboratories with high resolution mass spectrometry capabilities and, therefore, lead to the wider availability of serum cholesterol reference methods in the future.

## Conclusion

The selectivity of detection provided by high resolution mass spectrometry is a powerful tool in developing accurate analytical methods in clinical chemistry. In the case of prostanoid measurements, it is likely that GC/MS methods may be the only methods, which provide good analytical data. In the case of serum cholesterol measurements, GC/MS reference methods may lead to an improvement in routine assay by allowing more careful quality control and assurance, and more accurate calibration materials.

## Figures and Tables

**Table 1:** Cholesterol concentrations for NBS Certified Human Serum

Analysis set	Cholesterol concentration, mg/dL			
	#1	#2	ave	range
1	144.2	144.1	144.2	0.1
2	140.4	146.7	143.6	6.3
3	142.0	138.4	140.2	3.6
4	138.0	141.9	140.0	3.9
5	146.0	145.2	145.6	0.8
6	142.9	141.8	142.4	1.1
7	142.1	138.6	140.4	3.5
